# Editorial Comment: Prevalence of human papillomavirus DNA and p16INK4a in penile cancer and penile intraepithelial neoplasia: a systematic review and meta-analysis

**DOI:** 10.1590/S1677-5538.IBJU.2020.04.09

**Published:** 2020-05-20

**Authors:** Gustavo Cardoso Guimarães

**Affiliations:** 1 Departamento de Oncologia Cirúrgica Beneficência Portuguesa de São Paulo São PauloSP Brasil Chefe do Departamento de Oncologia Cirúrgica, Beneficência Portuguesa de São Paulo, São Paulo, SP, Brasil

Olesen TB ^1^, Sand FL^1^, Rasmussen CL ^1^, Albieri V ^2^, Toft B G^3^, Norrild B^4^, et al.

^1^Unit of Virus, Lifestyle, and Genes, Danish Cancer Society Research Centre, Copenhagen, Denmark; ^2^Unit of Statistics and Pharmacoepidemiology, Danish Cancer Society Research Centre, Copenhagen, Denmark ^3^ Department of Pathology, Rigshospitalet, Copenhagen, Denmark; ^4^Department of Cellular and Molecular Medicine, University of Copenhagen, Copenhagen, Denmark

Lancet Oncol. 2019 Jan;20(1):145-158

**DOI: 10.1016/S1470-2045(18)30682-X | ACCESS: 10.1016/S1470-2045(18)30682-X**

## COMMENT

In this paper, Tina Bech Olesens and colleagues, have assessed pooled HPV DNA prevalence in penile intraepithelial neoplasia or p16INK4a percent positivity in penile cancer and penile intraepithelial neoplasia and the prevalence of HPV DNA and p16INK4a positivity in penile cancer and penile intraepithelial neoplasia worldwide.

They made a systematic review and meta-analysis, in PubMed, Embase, and the Cochrane Library until July 24, 2017, for English-language articles published from Jan 1, 1986, onwards reporting the prevalence of HPV DNA and p16INK4a positivity, either alone or in combination, in at least five cases of penile cancer or penile intraepithelial neoplasia.

Using random-effects models, they estimated the pooled prevalence and 95% CI of HPV DNA and p16INK4a positivity in penile cancer and penile intraepithelial neoplasia, stratifying by histological subtype and HPV DNA or p16INK4a detection method. Type-specific prevalence of HPV6, HPV11, HPV16, HPV18, HPV31, HPV33, and HPV45 in penile cancer was estimated.

The authors searches identified 1836 non-duplicate records, of which 73 relevant papers (71 studies) were found to be eligible. The pooled HPV DNA prevalence in penile cancer (52 studies; n=4199) was 50·8%. A high pooled HPV DNA prevalence was seen in basaloid squamous cell carcinomas and in warty-basaloid carcinoma. The predominant oncogenic HPV type in penile cancer was HPV16 followed by HPV6 and HPV18. The pooled HPV DNA prevalence in penile intraepithelial neoplasia (19 studies; n=445) was 79·8%. The pooled p16INK4a percent positivity in penile cancer (24 studies; n=2295) was 41·6% (p <0·0001), with a high pooled p16INK4a percent positivity in HPV-related squamous cell carcinoma as compared with non-HPV-related squamous cell carcinoma. Moreover, among HPV-positive cases of penile cancer, the p16INK4a percent positivity was 79·6%, compared with 18·5% in HPV-negative penile cancers. The pooled p16INK4a percent positivity in penile intraepithelial neoplasia (six studies; n=167) was 49·5%.

In this interesting manuscript the authors concluded that a large proportion of penile cancers and penile intraepithelial neoplasias are associated with infection with HPV DNA (predominantly HPV16), emphasising the possible benefits of HPV vaccination in men and boys.

